# Dietary Inflammation Index and Its Association with Long-Term All-Cause and Cardiovascular Mortality in the General US Population by Baseline Glycemic Status

**DOI:** 10.3390/nu14132556

**Published:** 2022-06-21

**Authors:** Sheng Yuan, Chenxi Song, Rui Zhang, Jining He, Kefei Dou

**Affiliations:** 1 State Key Laboratory of Cardiovascular Disease, Beijing 102308, China; yuansheng@student.pumc.edu.cn (S.Y.); songchenxi@fuwaihospital.org (C.S.); zhangrui@fuwai.com (R.Z.); hejining95@163.com (J.H.); 2 Cardiometabolic Medicine Center, Department of Cardiology, Fuwai Hospital, National Center for Cardiovascular Diseases, Chinese Academy of Medical Sciences and Peking Union Medical College, Beijing 100037, China

**Keywords:** diabetes mellitus, prediabetes, dietary inflammation index, nutrition, inflammatory diet, NHANES

## Abstract

Dietary inflammatory potential has been proven to be correlated with the incidence of diabetes and cardiovascular diseases. However, the evidence regarding the impact of dietary inflammatory patterns on long-term mortality is scarce. This cohort study aims to investigate the dietary inflammatory pattern of the general US individuals by baseline glycemic status and to estimate its association with long-term mortality. A total of 20,762 general American adults with different glycemic statuses from the National Health and Nutrition Examination Survey were included. We extracted 24-h dietary information, and the dietary inflammatory index (DII) was calculated. The outcomes were defined as 5-year all-cause and cardiovascular mortality. Compared with the normoglycemia group, individuals with prediabetes and type 2 diabetes had higher DII scores (overall weighted *p* < 0.001). Compared with low DII scores, participants with high DII scores were at a higher risk of long-term all-cause mortality (HR: 1.597, 95% CI: 1.370, 1.861; *p* < 0.001) and cardiovascular mortality (HR: 2.036, 95% CI: 1.458, 2.844; *p* < 0.001). The results were stable after adjusting for potential confounders. Moreover, the prognostic value of DII for long-term all-cause mortality existed only in diabetic individuals but not in the normoglycemia or prediabetes group (*p* for interaction = 0.006). In conclusion, compared to the normoglycemia or prediabetes groups, participants with diabetes had a higher DII score, which indicates a greater pro-inflammatory potential. Diabetic individuals with higher DII scores were at a higher risk of long-term all-cause and cardiovascular mortality.

## 1. Introduction

Globally, the number of patients with diabetes and its devastating complications is increasing persistently in the past three decades, which is a major health threat to both developed and developing countries [1,2]. Due to the damage to the vascular smooth muscle cell and endothelial cell function [3], vascular diabetic complications cover almost all types of blood vessels and contribute to most of the mortality, hospitalization, and morbidity in patients with diabetes [4,5]. Obesity, decreased physical activity, population aging, and energy-dense diets are the primary causes of the rising diabetes rate [6]. Among those risk factors, the relationship between diabetes and nutrition or diet has received considerable attention [7,8,9,10].

Chronic inflammation plays a significant role in the etiology of diabetes [11,12]. Diet may interfere with the development of diabetes, which may be achieved through the influence of chronic inflammation. Many studies have demonstrated the correlation between pro-inflammatory food and diabetic risk [13,14]. A cross-sectional study of diabetes-free women revealed that red meat consumption was associated with elevated plasma inflammatory factors, fasting insulin, and glycated hemoglobin [13]. Moreover, an increasing number of studies have found that the Mediterranean diet, which was proven to have an anti-inflammatory effect [15,16,17], was associated with a lower diabetic risk [18,19,20,21].

The dietary inflammatory index (DII) was a dietary assessment tool developed based on the summary of published literature and aimed to estimate the inflammatory potential of an individual’s diet [22]. A high DII score, which was associated with elevated inflammatory markers, such as C-reactive protein (CRP), indicates a pro-inflammation diet and has been reported to be correlated with an increased risk of obesity, type 2 diabetes, and cardiovascular diseases [23,24,25,26,27]. Moreover, populations with higher DII scores were proven to have higher cardiovascular mortality [28,29]. However, currently, evidence about the relationship between DII and long-term mortality of subjects with different glycemic statuses is scarce. Therefore, our study aims to investigate the long-term prognostic value of DII among participants with normoglycemia, prediabetes, and type 2 diabetes, which may contribute to precise prognosis prediction and diabetes management.

## 2. Materials and Methods

### 2.1. Study Population

This cohort study was conducted following the Strengthening the Reporting of Observational Studies in Epidemiology (STROBE) reporting guideline for cohort studies [30]. The participants included in our analysis were extracted from the National Health and Nutrition Examination Survey (NHANES), a periodically conducted survey that obtains nationally-representative samples of the general Americans with a complex, multistage probability design [31]. In this study, we extracted participants from the 2007–2014 cycle. Adults with complete 24-h dietary data were included. The exclusion criteria included: (1) age <20 years old; (2) participants with pregnancy; and (3) participants without endpoint information.

### 2.2. Dietary Information

Dietary information was extracted from NHANES, which was collected through 24-h dietary recall interviews in the mobile examination center and was validated by the Nutrition Methodology Working Group [32]. Following the DII calculating protocol reported by N. Shivappa et al. [22], 28 food parameters in NHANES were used to calculate the DII, including carbohydrates, protein, total fat, alcohol, fiber, cholesterol, saturated fat, monounsaturated fatty acids (MUFAs), polyunsaturated fatty acids (PUFAs), niacin, vitamin A, thiamin, riboflavin, vitamin B6, vitamin B12, vitamin C, vitamin D, vitamin E, Fe, Mg, Zinc, Selenium, folic acid, β-carotene, caffeine, energy, n-3 fatty acids, and n-6 fatty acids. Previous studies have reported that DII calculated based on less than 30 food parameters kept its predictive ability [33,34].

A lower negative DII score suggests an anti-inflammatory effect, while a higher positive DII score means a pro-inflammatory effect of diet. According to the methods of N. Shivappa et al. [22], the DII calculation should be standardized to a world database that contains standard mean and standard deviation for each food parameter. The database was constructed by examining the relationship between parameters, including food components, and inflammation, in 1943 published articles. A parameter with proof of anti-inflammation effect would obtain a score of “−1”, while a food parameter would receive a score of “+1” if it was reported to be correlated with a reduced level of anti-inflammatory cytokines or increased level of proinflammatory cytokines. 

These values were further weighted according to the study design. For each included parameter, we first extracted the individualized consumption value and then subtracted it from the standard mean and divided this value by the standard deviation. To minimize the effect of right skewing, these values were converted to a centered percentile score. To achieve a symmetrical distribution with values centered around 0 and bounded between −1 and +1, each percentile score was doubled, and then we subtracted “1”. This centered percentile value for each food parameter was then multiplied by its corresponding inflammatory effect score to obtain the DII score for each food component. Finally, 28 food-specific DII scores were summed to create the overall DII score for each participant.

### 2.3. Diseases and Endpoint Definitions

Type 2 diabetes was defined as a self-reported physician diagnosis of diabetes, glycated hemoglobin A_1c_ (HbA_1c_) ≥ 6.5%, fasting glucose ≥ 7.8 mmol/L, or use of insulin or oral hypoglycemic medication. Prediabetes was defined as HbA1c 5.7%–6.4% (39–46 mmol/mol) or impaired fasting glucose (IFG) [fasting plasma glucose (FPG): 110–125 mg/dL (6.1–6.9 mmol/L)], or impaired glucose tolerance (IGT) [Oral glucose tolerance test 2-h glucose value ≥ 140 mg/dL (7.8 mmol/L) but < 200 mg/dL (11.1 mmol/L) and FPG < 126 (7.0 mmol/L)]. Hypertension was diagnosed as a self-reported physician diagnosis of hypertension, use of antihypertensive drugs, or systolic blood pressure ≥ 140 mmHg and/or diastolic blood pressure ≥ 90 mmHg (at least three times). 

Participants who met at least one of the following criteria were diagnosed with hyperlipidemia: (1) elevated total cholesterol ≥ 200 mg/dL (5.18 mmol/L); (2) high triglyceride level (≥150 mg/dL); (3) low density lipoprotein-cholesterol (LDL-c) ≥ 130 mg/dL (3.37 mmol/L); (4) high density lipoprotein-cholesterol (HDL-c) < 40 mg/dL (1.04 mmol/L) in men and 50 mg/dL (1.30 mmol/L) in women; and (5) use of cholesterol-lowering drugs. We set the time of follow-up time as 5 years. The primary endpoint of follow-up was all-cause death, which was extracted from the records of the National Death Index (NDI). The secondary endpoint was cardiovascular death, which was defined according to the International Classification of Diseases-10 codes (I00–I09, I11, I13, I20–I51) and was also extracted from NDI.

### 2.4. Statistics

To represent the overall US population, all analyses incorporated oversampling, clustering, and stratification as recommended by the NHANES data analysis guideline [31]. Continuous variables are listed as the weighted mean and 95% confidence interval (CI), while categorical variables are presented as weighted proportions. Basic characteristics are compared by baseline glycemic status using the adjusted Wald test for continuous variables and Rao-Scott χ^2^ test for categorical variables.

The weighted Cox proportional hazard regression models were adopted to assess the impact of DII on participants’ long-term mortality, which were adjusted for age, sex, educational level, BMI, smoke, hypertension, hyperlipidemia, glycemic status, recreational activity, and alcohol consumption. In addition to estimating DII as a continuous variable, we equally classified participants into three groups: low DII, medium DII, and high DII. Similar Cox regression models as well as weighted Kaplan-Meier curves were adopted to estimate the correlation between all-cause and cardiovascular mortality and different DII groups. 

Furthermore, to test whether the impact of DII on prognosis is different across patients with different glycemic statuses, the weighted Cox regression model and interaction *p* value were used to estimate the relationship between DII (continuous/categorical variable) and participants’ long-term mortality in participants with normal glucose status, prediabetes, and type 2 diabetes. The regression model was adjusted for age, sex, educational level, BMI, smoke, hypertension, hyperlipidemia, recreational activity, and alcohol consumption.

All analyses were conducted by the R software (version 4.1.2, R Foundation for Statistical Computing, Vienna, Austria) and Stata (version 16.0, StataCorp, College Station, TX, USA). A two-sided *p* value < 0.05 was considered statistically significant.

## 3. Results

### 3.1. Basic Characteristics by Baseline Glycemic Status

**Figure 1:**
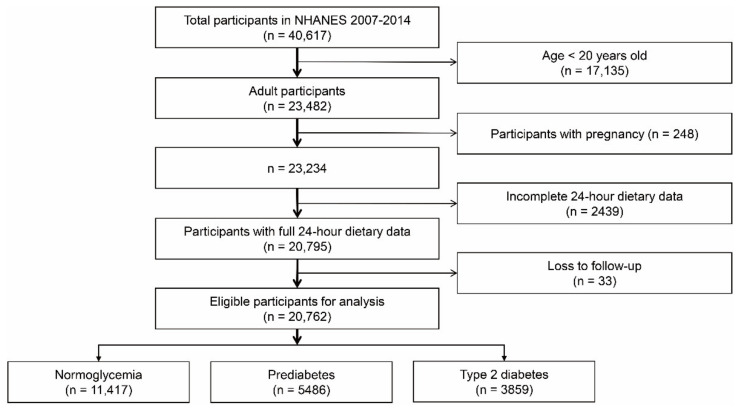
Flowchart of participant selection from NHANES database. NHANES: National Health and Nutrition Examination Survey.

Following the pre-specified inclusion and exclusion criteria, a total of 20,762 participants were included in our study, among which 3859 were diagnosed with type 2 diabetes, 5489 with prediabetes, and 11,417 with normal glucose status (Figure 1). Table 1 lists the comparison of basic characteristics by glycemic status. Many variables showed an increasing relationship among patients with normoglycemia, prediabetes, and type 2 diabetes, such as age, BMI, waist, systolic blood pressure, HbA_1c_, and triglycerides, which may indicate a worse health status in patients with prediabetes or type 2 diabetes. Similarly, we also found that patients with abnormal glucose metabolism were more likely to have a combination of hypertension or hyperlipidemia. 

Interestingly, compared with the normoglycemia group [113.94 (112.40,115.48) mg/dL], patients with prediabetes had a high level of LDL-c [122.04 (119.84, 124.24) mg/dL], while patients with type 2 diabetes had a better control of LDL-c [106.52 (103.97, 109.06) mg/dL]. As for the living habits, the percentage of former smokers was higher while the percentage of current smokers was lower in the diabetic population. The proportion of moderate or heavy drinking was also lower in participants with prediabetes or type 2 diabetes. Moreover, participants with type 2 diabetes were less likely to participate in recreational activity.

### 3.2. Comparison of DII Score by Baseline Glycemic Status

**Figure 2:**
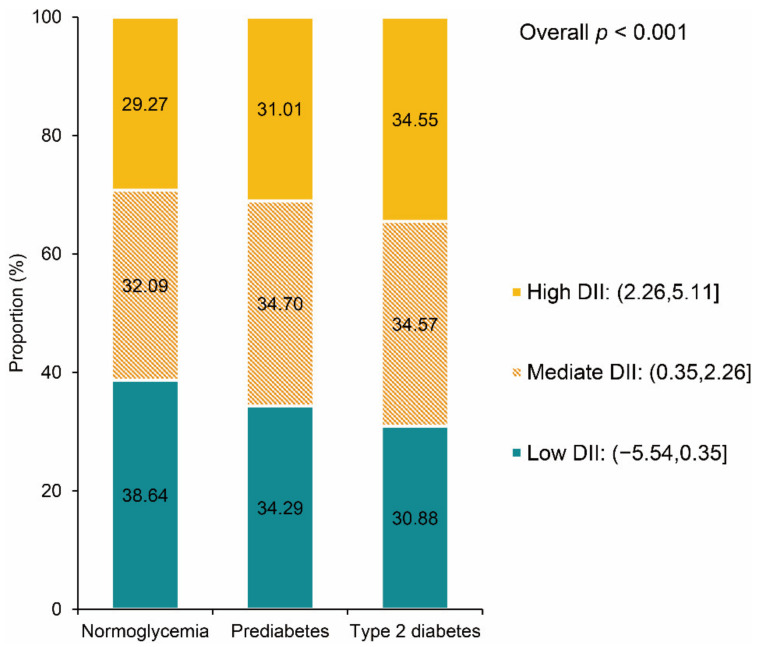
DII distribution by baseline glycemic status. DII: dietary inflammatory index.

Compared with the normoglycemia group (0.883, 95% CI: 0.793, 0.973), participants with prediabetes (1.081, 95% CI: 0.981, 1.181) and type 2 diabetes (1.249, 95% CI: 1.151, 1.346) had higher DII scores (overall weighted *p* < 0.001). Figure 2 presents the distribution of DII scores among three groups. The proportion of high DII scores was higher in participants with prediabetes or type 2 diabetes. Moreover, we compared the component of DII scores among the three groups to find the main cause of the difference. 

**Figure 3:**
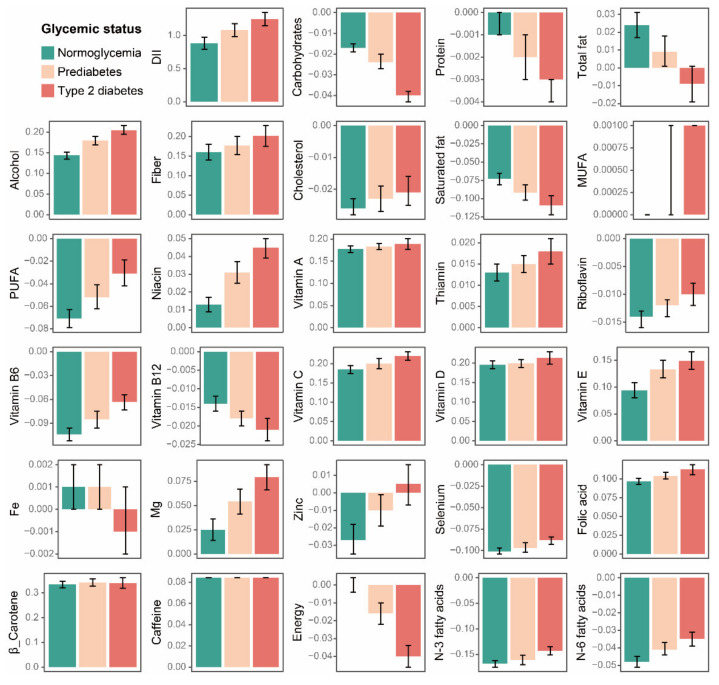
Comparison of the DII component scores by baseline glycemic status. Data are presented as the weighted mean value and 95%CI. DII: dietary inflammatory index; MUFA: monounsaturated fatty acids; and PUFA: polyunsaturated fatty acids.

Participants with type 2 diabetes had higher scores in alcohol, fiber, MUFA, PUFA, niacin, thiamin, riboflavin, vitamin B6, vitamin C, vitamin E, Mg, Zinc, Selenium, folic acid, N-3 fatty acids, and N-6 fatty acids (Figure 3, Table 2). We also noticed lower scores of participants with type 2 diabetes in certain components, such as carbohydrates, protein, total fat, saturated fat, vitamin B12, Fe, and energy. When compared to the normoglycemia group, the DII component scores remained consistent between participants with prediabetes and type 2 diabetes but to a lesser extent in the former.

### 3.3. Association between Dietary Inflammation and Long-Term Mortality

The overall weighted 5-year all-cause mortality was 4.56%, and the weighted 5-year cardiovascular mortality was 1.17%. The Cox regression models revealed that higher DII scores were associated with higher long-term all-cause mortality (HR per 1 score increase: 1.105, 95% CI: 1.065, 1.147; *p* < 0.001) and cardiovascular mortality (HR per 1 score increase: 1.172, 95% CI: 1.092, 1.258; *p* < 0.001) (Table S1 in the Supplementary Materials). 

**Figure 4:**
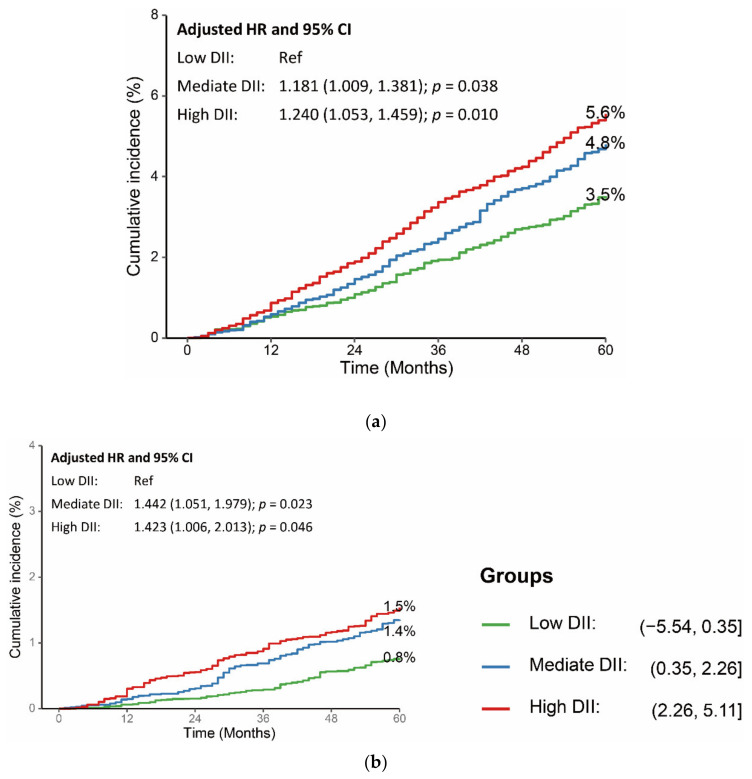
Association between DII scores and long-term (**a**) all-cause mortality and (**b**) cardiovascular mortality. HR was adjusted for age, sex, educational level, BMI, smoke, hypertension, hyperlipidemia, glycemic status, recreational activity, and alcohol consumption. CI: confidence interval; DII: dietary inflammatory index; HR: hazard ratio; and Ref: reference.

The association was stable after adjusting for age, sex, educational level, BMI, smoke, hypertension, hyperlipidemia, glycemic status, recreational activity, and alcohol consumption. Compared with participants with low DII scores, participants with mediate or high DII scores had higher risk of all-cause death (Mediate DII: adjusted HR: 1.181, 95% CI: 1.009, 1.381; *p* = 0.038; high DII: adjusted HR: 1.240, 95% CI: 1.053, 1.459; *p* = 0.010) and cardiovascular death (adjusted HR: 1.442, 95% CI: 1.051, 1.979; *p* = 0.023; high DII: adjusted HR: 1.423, 95% CI: 1.006, 2.013; *p* = 0.046) (Figure 4, Table S1 in the Supplementary Materials).

### 3.4. Dietary inflammation and Long-Term Mortality across Participants with Different Glycemic STATUSES

To estimate the impact of baseline glycemic status on the long-term prognostic value of DII, we performed adjusted Cox regression models in three groups: normoglycemia, prediabetes, and type 2 diabetes groups. As shown in Table 3, the association between DII scores and 5-year all-cause mortality was only significant in participants with type 2 diabetes (adjusted HR per 1 score increase 1.083, 95% CI: 1.014, 1.156; *p* = 0.017). DII was a better long-term all-cause mortality indicator in the type 2 diabetes group than was the normoglycemia or prediabetes group (*p* for interaction = 0.030). 

When treated as a categorical variable, a high DII score of participants with type 2 diabetes was associated with higher 5-year all-cause (adjusted HR 1.626, 95% CI: 1.208, 2.188; *p* = 0.001) and cardiovascular mortality (adjusted HR 1.980, 95% CI: 1.043, 3.761; *p* = 0.037) compared with low DII score. Participants with mediate DII scores in the type 2 diabetes group had a similar risk of long-term mortality. However, there was no significant correlation between DII and long-term mortality in the normoglycemia and prediabetes groups. The superiority of DII’s prognostic value for long-term all-cause mortality in the type 2 diabetes group over the normoglycemia or prediabetes group was robust. (Continuous DII: *p* for interaction = 0.030; categorical DII: *p* for interaction = 0.006)

## 4. Discussion

Our study included a total of 20,762 participants, which represented 218,988,071 of the general US population, and we found that prediabetic or diabetic participants had a more pro-inflammatory diet compared with the normoglycemia group. Participants with mediate or higher DII scores were at higher risk of long-term all-cause and cardiovascular mortality. The prognostic effect of DII was only significant in diabetic participants and not in the prediabetic or normoglycemia group.

Many studies have shown that certain diet patterns, such as advanced glycation end products (AGEs), antioxidant diet, and the Mediterranean diet, can affect the low-level inflammation or body composition, and thus influence the incidence and development of some chronic diseases [15,35,36]. Previous research has suggested that dietary patterns may influence the incidence of diabetes. An analysis of 200,727 US participants from three prospective cohort studies conducted over 20 years revealed that eating more healthy plant foods and eating fewer animal foods was associated with a 20% reduction in diabetic risk [37].

A 20-year prospective cohort of 70,991 women discovered that a higher anti-inflammatory diet (as measured by DII) was linked to a reduced risk of type 2 diabetes [26]. Our study confirmed this relationship and found a sequentially increasing DII score across the normoglycemia, prediabetes, and type 2 diabetes groups. Moreover, component analysis in our results revealed that participants with prediabetes or type 2 diabetes had higher scores in alcohol, fiber, MUFA, PUFA, niacin, thiamin, riboflavin, vitamin B6, vitamin C, vitamin E, Mg, Zinc, Selenium, folic acid, N-3 fatty acids, and N-6 fatty acids compared with participants with normoglycemia.

Interestingly, diabetic participants had lower scores in some key nutritional indicators, such as carbohydrates, protein, total fat, saturated fat, and energy. This dietary pattern may come from the active adjustment after the diagnosis of prediabetes or diabetes. Another study based on the NHANES database discovered that participants with diagnosed prediabetes or diabetes were more likely to be concerned about nutrition fact labels when making daily food purchases [38].

However, rather than simple calorie calculations, we should be concerned about the complex and long-term influences of different foods on health [39]. Nutrition science found that overall dietary patterns and specific foods, instead of single isolated nutrients were more important for cardiometabolic health [40,41]. In participants with prediabetes or diabetes, a shortage of vitamins, critical micronutrients, and unsaturated fatty acids, as shown in our study, may lead to poor health and disease progression, which requires attention in diabetic care.

Dietary patterns are linked not only to the occurrence of chronic diseases but also to disease prognosis. A meta-analysis of 14 research articles found that individuals in the highest DII group had a higher risk of cardiovascular disease incidence as well as cardiovascular mortality [42]. Park et al. estimated the relationship between dietary inflammatory potential and prognosis in participants with different metabolic phenotypes [34]. They included 3733 adults from the NHANES III database (1988–1994) and revealed that the DII score was correlated with elevated all-cause and cardiovascular mortality in individuals with metabolically unhealthy obesity, which has not been observed in metabolically healthy obese individuals.

The target population of our study consists of 20,762 participants who participated in the NHANES project in the near twenty years (2007–2014). Similarly, our results demonstrated that a higher DII score was associated with higher long-term all-cause and cardiovascular mortality in participants with type 2 diabetes. The correlation was not identified in the prediabetes or normoglycemia group. Our findings imply that dietary inflammatory potential has a major influence on the long-term prognosis of diabetic patients, a topic that requires further attention in diabetes management.

To our knowledge, this is the first work that compares the long-term prognostic value of DII in the general American participants by baseline glycemic status. There are several limitations to our study. First, DII was calculated from self-reported dietary data, and recall bias was inevitable. Secondary, we extracted the 24 h dietary information to represent the daily pattern, which may change over time. Second, the DII used in our study was calculated from 28 food parameters due to the limitation of dietary data in the NHANES database. Previous studies have reported that DII calculated based on less than 30 food parameters kept its predictive ability [33,34]. 

Thirdly, we discovered that participants with prediabetes had higher LDL-c levels than the normoglycemia group, whereas patients with type 2 diabetes had better LDL-c control. This phenomenon could be explained by the fact that people with diabetes are more likely to visit the hospital and undergo laboratory tests, allowing their complications, such as hyperlipidemia, to be better managed.

However, this is our hypothesis, and because therapy data is limited, a specific reason should be investigated in future research. Finally, although we adjusted the potential risk factors including age, sex, body mass index, smoke, hypertension, educational level, hyperlipidemia, glycemic status, recreational activity, and moderate or heavy drinker in the multivariable Cox regression models, cardiovascular pathology and medication therapy were not involved due to the limitation of database, which may have an important impact on the cardiovascular mortality.

## 5. Conclusions

Our study identified a more pro-inflammatory diet in the diabetic participants compared with the general Americans. Participants with a higher DII score were at higher risk of 5-year all-cause and cardiovascular mortality. The prognostic value of DII existed only in individuals with type 2 diabetes but not in the normoglycemia or prediabetes group. The result calls for a comprehensive assessment of the dietary inflammatory potential in diabetic patients. Moreover, whether an anti-inflammatory dietary adjustment can improve the long-term prognosis of diabetes should be assessed in future trials.

## Figures and Tables

**Table 1:** Basic characteristics of participants by baseline glycemic status.

	Total (*n* = 20,762)	Normoglycemia (*n* = 11,417)	Prediabetes (*n* = 5486)	Type 2 Diabetes (*n* = 3859)	*p*
Age (years)	47.35 (46.78, 47.92)	42.06 (41.37,42.76)	54.20 (53.59,54.81)	59.58 (58.98,60.18)	<0.001
Male	48.70	48.62	47.86	50.36	0.250
BMI (kg/m^2^)	28.81 (28.64, 28.98)	27.47 (27.28,27.65)	30.01 (29.71,30.31)	32.90 (32.52,33.27)	<0.001
Waist (cm)	98.62 (98.18, 99.05)	94.80 (94.26, 95.33)	102.14 (101.56,102.72)	109.93 (109.15,110.71)	<0.001
Ethnicity					<0.001
Non-Hispanic white	67.92	70.28	65.65	61.06	
Non-Hispanic black	11.34	9.53	13.75	15.38	
Mexican American	8.45	8.22	8.28	9.79	
Other Hispanic	5.47	5.45	5.17	6.09	
Other race	6.83	6.52	7.15	7.68	
Educational level					<0.001
Less than high school	17.45	14.40	20.29	26.48	
High school or equivalent	22.66	20.91	25.23	26.16	
College or above	59.59	64.69	54.48	47.36	
Smoke					<0.001
Never smoker	54.50	57.28	50.67	48.49	
Former smoker	24.42	21.20	27.00	34.67	
Current smoker	21.07	21.52	22.33	16.83	
Moderate or heavy drink	36.25	42.27	28.96	21.47	<0.001
Recreational activity					<0.001
No	46.62	40.55	52.41	64.24	
Moderate	28.56	27.45	31.72	28.10	
Vigorous	24.83	32.00	15.87	7.66	
SBP (mmHg)	122.42 (121.95,122.89)	119.21 (118.76,119.67)	126.19 (125.43,126.95)	130.57 (129.56,131.58)	<0.001
DBP (mmHg)	70.93 (70.49,71.37)	70.88 (70.39,71.38)	71.81 (71.32,72.30)	69.56 (68.79,70.33)	<0.001
Hypertension	36.67	25.62	47.14	68.96	<0.001
Hyperlipidemia	71.07	62.55	82.76	88.60	<0.001
HbA1c (%)	5.62 (5.60,5.64)	5.23 (5.22,5.24)	5.77 (5.76,5.79)	7.08 (7.01,7.15)	<0.001
Total cholesterol (mg/dL)	195.10 (194.12,196.08)	193.73 (192.50,194.96)	202.88 (201.21,204.55)	187.79 (185.72,189.85)	<0.001
HDL-c (mg/dL)	52.74 (52.32, 53.16)	54.50 (54.01,55.00)	51.32 (50.65,52.00)	47.11 (46.42,47.80)	<0.001
LDL-c (mg/dL)	115.12 (113.92,116.32)	113.94 (112.40,115.48)	122.04 (119.84,124.24)	106.52 (103.97,109.06)	<0.001
Triglycerides (mg/dL)	129.91 (126.76,133.06)	113.14 (109.94,116.35)	139.14 (133.90,144.38)	168.63 (159.26,178.00)	<0.001

BMI: body mass index; DBP: diastolic blood pressure; DII: dietary inflammatory index; HDL-c: high-density lipoprotein cholesterol; LDL-c: low-density lipoprotein cholesterol; and SBP: systolic blood pressure.

**Table 2:** Comparison of the components of DII by baseline glycemic status.

	Total (*n* = 20,762)	Normoglycemia (*n* = 11,417)	Prediabetes (*n* = 5486)	Type 2 diabetes (*n* = 3859)	*p*
DII	0.980 (0.908, 1.052)	0.883 (0.793, 0.973)	1.081 (0.981, 1.181)	1.249 (1.151, 1.346)	<0.001
Carbohydrates	−0.022 (−0.023, −0.020)	−0.017 (−0.019, −0.015)	−0.024 (−0.027, −0.020)	−0.040 (−0.043, −0.038)	<0.001
Protein	−0.001 (−0.002, −0.001)	−0.001 (−0.001, 0.000)	−0.002 (−0.003, −0.001)	−0.003 (−0.004, −0.003)	<0.001
Total fat	0.016 (0.011, 0.021)	0.024 (0.017, 0.031)	0.009 (0.001, 0.018)	−0.009 (−0.019, 0.001)	<0.001
Alcohol	0.161 (0.154, 0.168)	0.144 (0.135, 0.152)	0.180 (0.169, 0.190)	0.205 (0.195, 0.216)	<0.001
Fiber	0.170 (0.154, 0.186)	0.160 (0.140, 0.180)	0.177 (0.154, 0.201)	0.202 (0.175, 0.228)	0.025
Cholesterol	−0.024 (−0.026, −0.022)	−0.026 (−0.028, −0.023)	−0.023 (−0.027, −0.019)	−0.021 (−0.025, −0.016)	0.2852
Saturated fat	−0.082 (−0.089, −0.076)	−0.073 (−0.081, −0.065)	−0.092 (−0.102, −0.081)	−0.109 (−0.122, −0.096)	<0.001
MUFA	0.000 (0.000, 0.001)	0.000 (0.000, 0.000)	0.000 (0.000, 0.001)	0.001 (0.001, 0.001)	0.001
PUFA	−0.061 (−0.067, −0.055)	−0.071 (−0.079, −0.063)	−0.052 (−0.062, −0.041)	−0.031 (−0.042, −0.019)	<0.001
Niacin	0.022 (0.019, 0.025)	0.013 (0.009, 0.017)	0.031 (0.025, 0.037)	0.045 (0.039, 0.050)	<0.001
Vitamin A	0.180 (0.174, 0.187)	0.178 (0.170, 0.185)	0.183 (0.176, 0.190)	0.189 (0.177, 0.201)	0.170
Thiamin	0.014 (0.012, 0.015)	0.013 (0.011, 0.015)	0.015 (0.013, 0.017)	0.018 (0.015, 0.021)	0.020
Riboflavin	−0.013 (−0.014, −0.012)	−0.014 (−0.016, −0.013)	−0.012 (−0.014, −0.011)	−0.010 (−0.012, −0.008)	0.001
Vitamin B6	−0.094 (−0.100, −0.088)	−0.104 (−0.112, −0.096)	−0.085 (−0.096, −0.075)	−0.063 (−0.073, −0.054)	<0.001
Vitamin B12	−0.016 (−0.017, −0.014)	−0.014 (−0.016, −0.012)	−0.018 (−0.020, −0.016)	−0.021 (−0.024, −0.018)	<0.001
Vitamin C	0.193 (0.185, 0.202)	0.185 (0.174, 0.195)	0.200 (0.187, 0.213)	0.220 (0.209, 0.231)	<0.001
Vitamin D	0.198 (0.191, 0.206)	0.195 (0.185, 0.205)	0.199 (0.188, 0.209)	0.213 (0.197, 0.229)	0.174
Vitamin E	0.111 (0.101, 0.121)	0.094 (0.080, 0.108)	0.133 (0.117, 0.150)	0.149 (0.133, 0.166)	<0.001
Fe	0.001 (0.000, 0.002)	0.001 (0.000, 0.002)	0.001 (0.000, 0.002)	−0.001 (−0.002, 0.001)	0.027
Mg	0.040 (0.031, 0.049)	0.025 (0.014, 0.036)	0.054 (0.041, 0.067)	0.079 (0.066, 0.092)	<0.001
Zinc	−0.018 (−0.025, −0.011)	−0.027 (−0.035, −0.018)	−0.010 (−0.019, −0.001)	0.005 (−0.007, 0.016)	<0.001
Selenium	−0.098 (−0.101, −0.096)	−0.101 (−0.104, −0.097)	−0.097 (−0.102, −0.091)	−0.088 (−0.093, −0.084)	<0.001
Folic acid	0.101 (0.098, 0.104)	0.097 (0.093, 0.101)	0.104 (0.100, 0.109)	0.113 (0.106, 0.119)	<0.001
β-Carotene	0.337 (0.326, 0.348)	0.335 (0.321, 0.348)	0.342 (0.327, 0.357)	0.340 (0.319, 0.361)	0.739
Caffeine	0.084 (0.084, 0.084)	0.084 (0.084, 0.084)	0.084 (0.084, 0.084)	0.084 (0.084, 0.084)	0.367
Energy	−0.009 (−0.012, −0.006)	0.000 (−0.004, 0.004)	−0.016 (−0.022, −0.010)	−0.040 (−0.046, −0.034)	<0.001
N-3 fatty acids	−0.163 (−0.168, −0.158)	−0.168 (−0.175, −0.162)	−0.161 (−0.170, −0.152)	−0.143 (−0.151, −0.135)	<0.001
N-6 fatty acids	−0.044 (−0.047, −0.042)	−0.048 (−0.051, −0.045)	−0.041 (−0.044, −0.037)	−0.035 (−0.039, −0.031)	<0.001

The data are presented as the mean and 95% confidence interval. DII: Dietary inflammation index; MUFA: monounsaturated fatty acids; PUFA: polyunsaturated fatty acids.

**Table 3:** Association between DII and the long-term mortality of participants by baseline glycemic status.

	Normoglycemia (*n* = 11,417)	Prediabetes (*n* = 5486)	Type 2 Diabetes (*n* = 3859)	*p* for Interaction
All-cause mortality				
Continuous, per 1 score	1.004 (0.937, 1.075); *p* = 0.919	1.047 (0.967, 1.133); *p* = 0.255	1.083 (1.014, 1.156); *p* = 0.017	0.030
1st tertile (−5.54, 0.35]	Ref	Ref	Ref	0.006
2nd tertile (0.35, 2.26]	1.119 (0.834, 1.502); *p* = 0.455	0.878 (0.640, 1.204); *p* = 0.418	1.683 (1.266, 2.237); *p* < 0.001	
3rd tertile (2.26, 5.11]	1.011 (0.728, 1.404); *p* = 0.947	1.210 (0.848, 1.726); *p* = 0.295	1.626 (1.208, 2.188); *p* = 0.001	
Cardiovascular mortality				
Continuous, per 1 score	1.102 (0.958, 1.269); *p* = 0.175	1.032 (0.900, 1.184); *p* = 0.654	1.104 (0.962, 1.265); *p* = 0.158	0.867
1st tertile (−5.54, 0.35]	Ref	Ref	Ref	0.455
2nd tertile (0.35, 2.26]	1.548 (0.978, 2.449); *p* = 0.062	0.986 (0.527, 1.845); *p* = 0.965	1.864 (1.036, 3.353); *p* = 0.038	
3rd tertile (2.26, 5.11]	1.476 (0.725, 3.007); *p* = 0.283	0.934 (0.501, 1.739); *p* = 0.829	1.980 (1.043, 3.761); *p* = 0.037	

The data are presented as the adjusted HR and 95% CI. The Cox regression models are adjusted for age, sex, body mass index, smoke, hypertension, educational level, hyperlipidemia, recreational activity, and moderate or heavy drinker.

## Data Availability

All data are available at NHANES website https://www.cdc.gov/nchs/nhanes/index.htm (accessed on 1 June 2022).

## Supplementary Materials

The following supporting information can be downloaded at: https://www.mdpi.com/article/10.3390/nu14132556/s1, Table S1: Survival analysis of the relationship between DII scores and long-term mortality.
