# Supplementary material for: Dietary Inflammation Index and Its Association with Long-Term All-Cause and Cardiovascular Mortality in the General US Population by Baseline Glycemic Status
Source: Nutrients. 2022 Jun 21;14(13):2556. doi: 10.3390/nu14132556 (PMC9268060; doi:10.3390/nu14132556)
Supplement: Supplementary file 1 [file nutrients-14-02556-s001.zip › nutrients-1777222-supplementary.pdf]

Table S1. Survival analysis of the relationship between DII scores and long-term mortality

	Crude HR (95%CI)	*p*1	Adjusted HR (95%CI)*	*p*2
**All-cause mortality**				
Per 1 score increase	1.105 (1.065, 1.147)	< 0.001	1.043 (1.005, 1.082)	0.026
Low DII: (-5.54, 0.35]	Ref		Ref	
Mediate DII: (0.35, 2.26]	1.373 (1.167, 1.614)	< 0.001	1.181 (1.009, 1.381)	0.038
High DII: (2.26, 5.11]	1.597 (1.370, 1.861)	< 0.001	1.240 (1.053, 1.459)	0.010
**Cardiovascular mortality**				
Per 1 score increase	1.172 (1.092, 1.258)	< 0.001	1.080 (1.005, 1.160)	0.037
Low DII: (-5.54, 0.35]	Ref		Ref	
Mediate DII: (0.35, 2.26]	1.780 (1.299, 2.438)	< 0.001	1.442 (1.051, 1.979)	0.023
High DII: (2.26, 5.11]	2.036 (1.458, 2.844)	< 0.001	1.423 (1.006, 2.013)	0.046

*Adjusted for age, sex, educational level, BMI, smoke, hypertension, hyperlipidemia, glycemic status, recreational activity, and alcohol consumption.

BMI: body mass index; CI: confidence interval; DII: Dietary inflammation index; HR: hazard ratio.
